# Adjuvant transarterial chemoembolization after radical resection contributed to the outcomes of hepatocellular carcinoma patients with high-risk factors

**DOI:** 10.1097/MD.0000000000007426

**Published:** 2017-08-18

**Authors:** Zhendong Gao, Gang Du, Yuguang Pang, Zhihao Fu, Chongzhong Liu, Yi Liu, Binghai Zhou, Du Kong, Binyao Shi, Zhengcheng Jiang, Bin Jin

**Affiliations:** a Department of General Surgery, Qilu Hospital of Shandong University; b School of Medicine, Shandong University, Jinan; c Department of General Surgery, Renmin Hospital of Lingcheng, Dezhou, China.

**Keywords:** disease-free survival, hepatocellular carcinoma, overall survival, radical resection, recurrence, transarterial chemoembolization

## Abstract

We aim to investigate the effects of postoperative adjuvant transarterial chemoembolization (TACE) on survival and recurrence in hepatocellular carcinoma (HCC) patients after radical resection. A total of 320 HCC patients underwent radical resection between January 2010 and January 2014 in Qilu Hospital, Shandong University were divided into 4 groups according to the frequency of postoperative adjuvant TACE. Patients were further stratified into subgroups (tumor diameter ≤5 or >5 cm) with low or high risk factors for recurrence or death. A low risk factor for recurrence or death was defined as Edmondson grade I/II without microvascular invasion (MiVI), while a high risk factor was defined as Edmondson grade III/IV or with MiVI. Survival data and recurrence rates were compared using the Kaplan–Meier method. Uni- and multivariate analyses were based on the Cox proportional analysis. Compared to those received no TACE, patients underwent 2 (log-rank, χ^2^ = 9.054, *P* = .003) or 3 (log-rank, χ^2^ = 4.228, *P* = .04) TACE showed delayed recurrence. Patients received 2 or 3 TACE showed extended overall survival (OS) compared with the other patients. No statistical differences were found between all the disease-free survival (DFS) and OS in low-risk subgroups. In the patients of the high-risk subgroup with a tumor diameter of ≤5, those received 2 TACE showed delayed recurrence compared with those received no TACE, and TACE (twice or thrice) can improve OS. For those of the high-risk subgroup with a tumor diameter of >5, TACE (twice or thrice) can delay recurrence and improve OS. Adjuvant TACE (twice or thrice) after radical resection is beneficial for HCC patients with poor differentiation and MiVI, especially for those with a tumor diameter of >5 cm.

## 1 Introduction

Hepatocellular carcinoma (HCC), leading to 500,000 deaths annually worldwide,^[1,2]^ is considered as the 2nd leading cause of cancer-related death.^[3]^ Nowadays, hepatectomy has been commonly used in the radical therapy for HCC^[4,5]^; however, a higher incidence of recurrence may present after radical hepatectomy within 5 years.^[6]^

In clinical practice, multiple adjuvant and neoadjuvant therapies have been commonly used in order to reduce recurrence and improve the overall survival (OS).^[7–10]^ Among these methods, transcatheter arterial chemoembolization (transarterial chemoembolization, TACE) is regarded as an adjuvant way to reduce the recurrence after hepatectomy. Nevertheless, the treatment efficiency of TACE for HCC after radical hepatectomy still remains controversial,^[11–13]^ and no consensus has been obtained on the proper time for postoperative TACE for patients with high or low risk factors. In this study, we designed a retrospective study on patients underwent radical hepatectomy with or without TACE to investigate the effects of postoperative adjuvant TACE on survival and recurrence in HCC patients after radical resection.

## 2 Materials and methods

### 2.1 Ethical approval

This study protocols were approved by the Ethical Committee of the Qilu hospital, Shandong University. The study was in line with the Declaration of Helsinki. Written informed consent was obtained from each patient before surgery.

### 2.2 Patients

**Figure 1:**
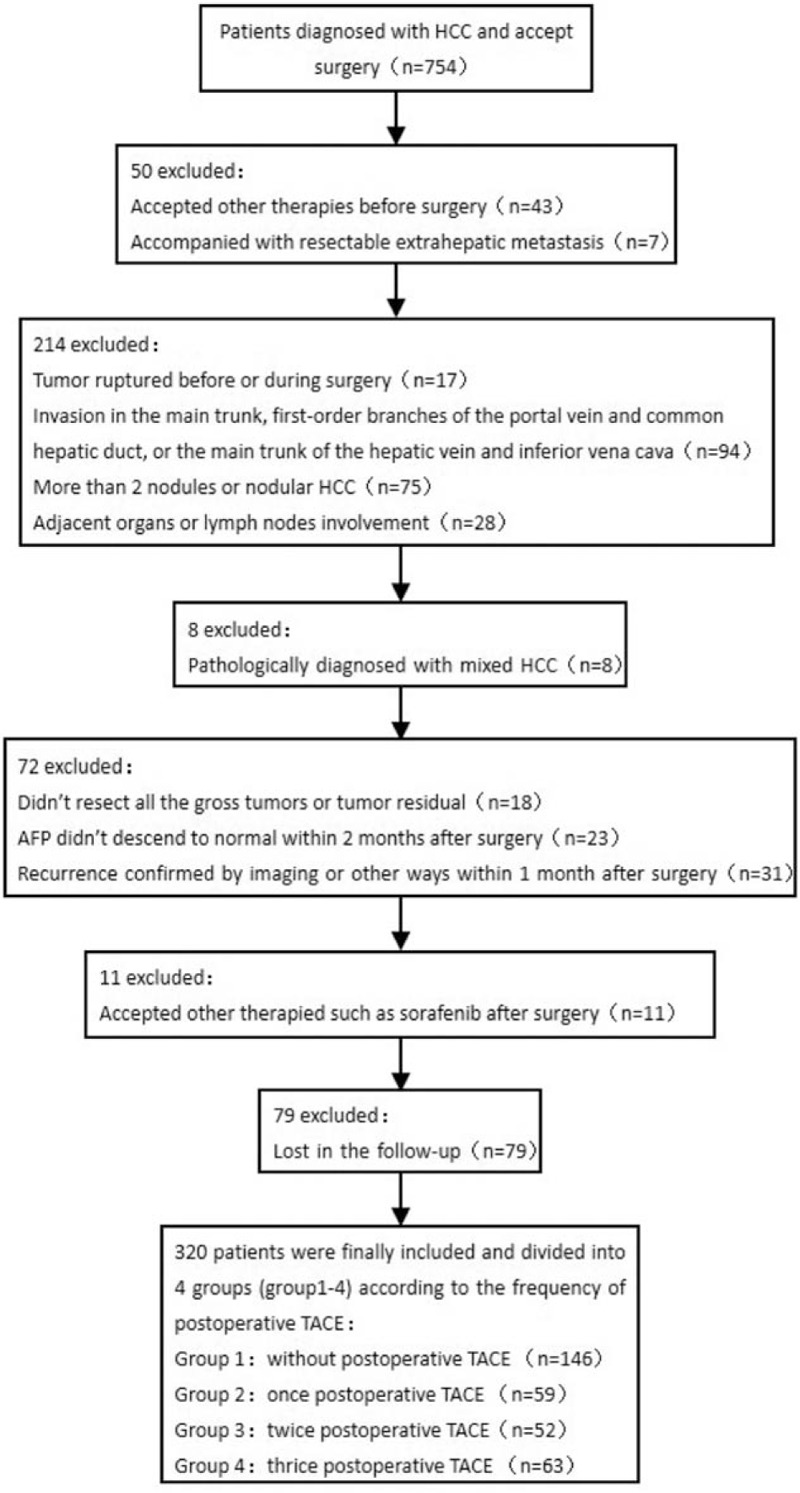
Flow chart of the study. A total of 754 patients who were diagnose with HCC and underwent surgery in our hospital between January 2010 and January 2014 were screened. A total of 434 patients were excluded, 320 patients considered suitable for this retrospective study were finally included and they are further divided into different groups according to the frequency of postoperative TACE. HCC = hepatocellular carcinoma, TACE = transarterial chemoembolization.

In this retrospective analysis, we collected the medical records of 754 patients diagnosed with HCC received surgery in our hospital between January 2010 and January 2014. In total, 434 patients were excluded and 320 patients were finally included (Fig. 1). The inclusion criteria were as follows: HCC patients with less than 3 nodules received no treatment before this study; those with no history of malignancy; those with no invasion of cancer cells in the main trunk, 1st-order branches of the portal vein and common hepatic duct, or the main trunk of the hepatic vein and inferior vena cava; those with no lymph node involvement; those with no intra- or extrahepatic metastasis, or satellite nodules; and those underwent radical resection of tumor lesions, defined as complete macroscopic removal of the tumor with negative histologic resection margins. The exclusion criteria were as follows: those with a preoperative liver function of grade C according to Child–Pugh scoring system; those lost in the follow-up; those with residual tumor or portal tumor thromboses in postsurgical imaging; and with elevation of alpha-fetoprotein (AFP) within 2 months after operation.

### 2.3 Grouping

Patients were categorized into 4 groups according to the frequency of TACE, including group 1, received no TACE and group 2 to 4 received 1, 2, and 3 TACE, respectively. Patients with a tumor diameter of more than 5 cm were recommended to receive TACE therapy at 1st, 2nd, and 3rd month after radical hepatectomy based on their own intentions. For those with a tumor diameter of less than 5 cm, postoperative TACE was performed based on patients’ own intentions.

### 2.4 Postoperative TACE

About 1 month after radical resection, a hepatic arterial catheter was placed into the proper hepatic artery through the femoral artery using the Seldinger technique after the recovery of liver function. Angiography was performed for the entire remnant liver to detect the staining of cancer cells, and a standard TACE was performed to each patient. For the chemotherapy, 0.5 g Floxuridine containing 50 to 100 mg lobaplatin or oxaliplatin, 30 to 50 mg doxorubicin, and 5 to 10 mL of Lipiodol was given to each patient.

### 2.5 Follow-up

The patients were followed up regularly and were monitored prospectively for recurrence and death. The disease-free survival (DFS) was calculated as the date of surgery to the recurrence, and the OS was calculated as the date of surgery to the date of death. In each follow-up visits, the liver function was determined together with the serum AFP quantification and ultrasound or contrast CT images. The patients were followed up every 1 to 2 months during the 1st postoperative year and 3 to 6 months thereafter. The recurrence was confirmed based on cytological/histological evidence or on the noninvasive diagnostic criteria for HCC.^[14]^ The patients with recurrence were treated with surgical resection, liver transplantation, percutaneous radiofrequency ablation, systematic chemotherapy or sorafenib, and TACE.

### 2.6 Statistical analysis

The statistical analysis was performed using SPSS 21.0 software (SPSS, Chicago, IL). The continuous variables were expressed as mean ± standard deviation (SD) or median (range). Categorical variables were expressed as specific number. The categorical variables were compared by the Chi square test or Fisher exact test, and the continuous variables by the ANOVA. Chi square test was used in presence of sample size of ≥40 and a theoretical frequency of ≥5. Fisher exact test was used in presence of sample size of less than 40 or a theoretical frequency of less than 1. Kaplan–Meier method was used to evaluate the survival of the patients. Log-rank test was used to evaluate the differences between the groups. Uni- and multivariate analyses were based on the Cox proportional analysis. *P* < .05 was considered as statistically significant.

## 3 Results

### 3.1 Patient characteristics

A total of 320 patients were included in this study, and the clinical characteristics were summarized in Table 1. Among these patients, 146 (54.46 ± 1.65 years) received no TACE (group 1), 59 (53.33 ± 2.07 years) received TACE once (group 2), 52 (55.61 ± 2.06 years) received TACE twice (group 3), and 63 (52.92 ± 2.99 years) received TACE thrice (group 4) before subgroup analysis. No statistical differences were noticed in the tumor characteristics, such as diameter, number of tumors, AFP level, microvascular invasion (MiVI), and Edmonson grade, among the 4 groups. Meanwhile, no significant difference was noticed in hepatitis B antigen positivity and liver cirrhosis, as well as total bilirubin, albumin, alanine aminotransferase level, aspartate aminotransferase level, gamma-glutamyl transpeptidase level, alkaline phosphatase level, platelet count, prothrombin time, or Child–Pugh classification. Moreover, no statistical differences were noticed in the types of surgical procedures among the patients of the 4 groups.

**Table 1:**
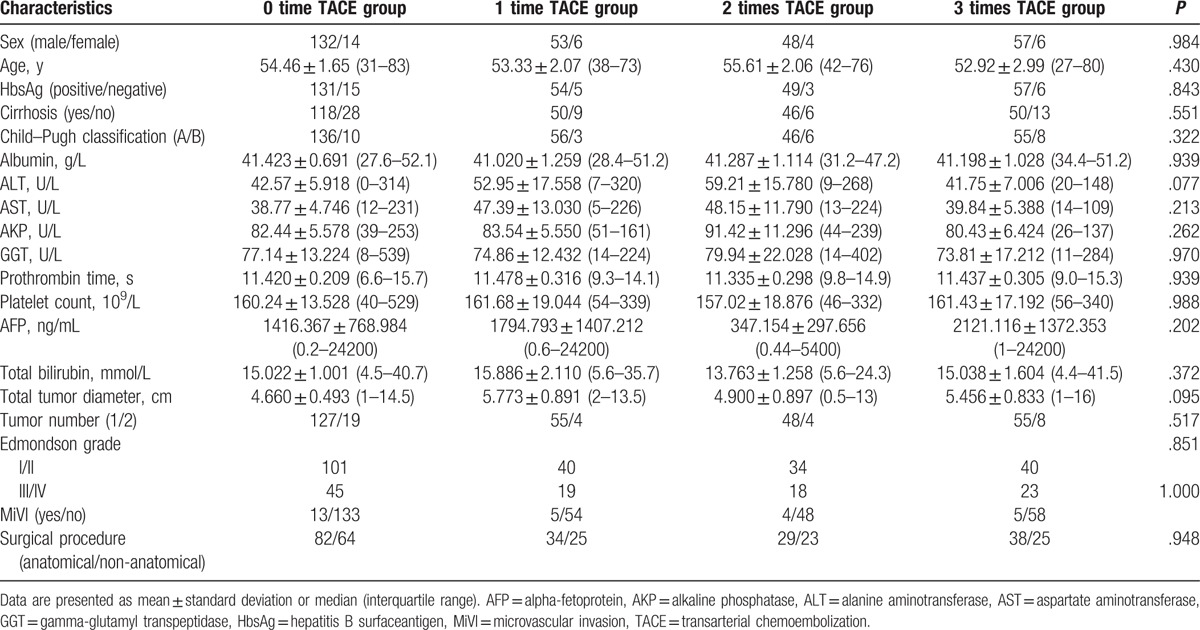
Clinical characteristics: baseline clinical characteristics of hepatocellular carcinoma patients treated by curative resection alone (0 time TACE) or by radical resection followed by 1 to 3 times TACE.

### 3.2 Recurrence and OS before subgroup analysis

**Figure 2:**
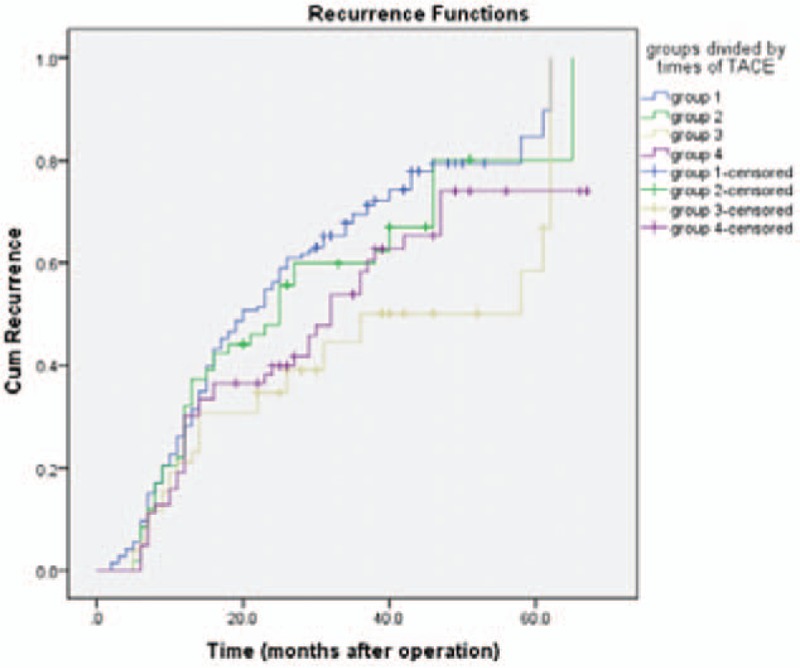
Recurrence curves for patients. Compared with group 1, remarkable improvement was noticed in the recurrence in group 3 (*P* = .003) and group 4 (*P* = .04).

The study was censored on May 31, 2016. The median follow-up period was 35 months (95% CI, 23.0–38.7 months) for group 1, 33 months for group 2, 39 months for group 3, and 45 months for group 4, respectively. In group 1, the 1-, 3-, and 5-year DFS was 72%, 30%, and 15%, respectively. In group 2, the 1-, 3-, and 5-year DFS was 68%, 40%, and 20%, respectively. In group 3, the 1-, 3-, and 5-year DFS was 79%, 50%, and 41%, while for the group 4 was 70%, 42%, and 26%, respectively. Pairwise comparison showed statistical differences were noticed in the cumulative incidences of recurrence between the patients in group 1 and group 3 (log-rank, χ^2^ = 9.054, *P* = .003). Meanwhile, remarkable difference was noticed in the recurrence in patients in group 1 and group 4 (log-rank, χ^2^ = 4.228, *P* = .04, Fig. 2).

**Figure 3:**
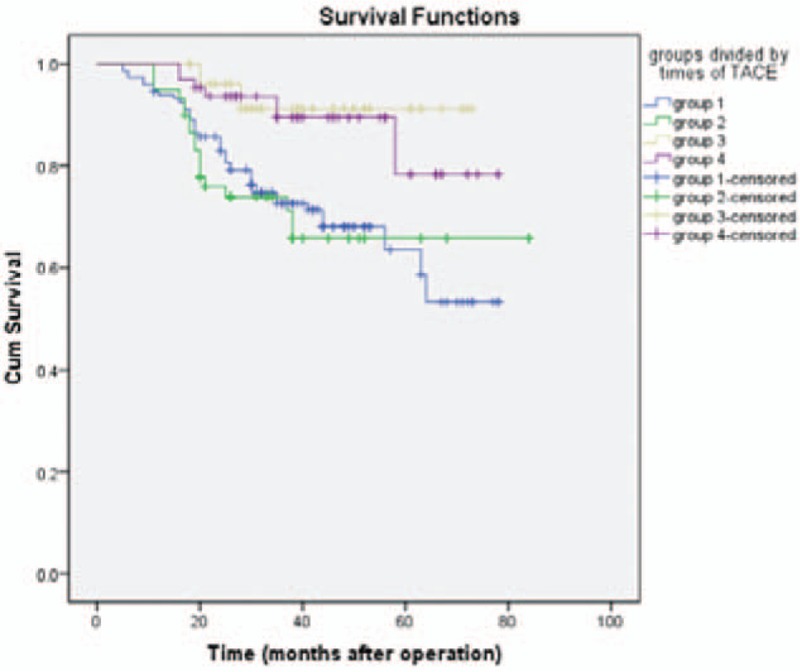
Survival curve for patients. Compared with group 1, remarkable elevation was noticed in the overall survival (OS) in group 3 (*P* = .002) and group 4 (*P* = .03).

The 1-, 3-, and 5-year OS in group 1 were 94%, 72%, and 63%, respectively. In group 2, the 1-, 3-, and 5-year OS was 95%, 74%, and 65%, respectively. In group 3, the 1-, 3-, and 5-year OS was 100%, 91%, and 91%, while in group 4, the 1-, 3-, and 5-year OS was 100%, 89%, and 78%, respectively. Compared with the group 1, remarkable increase was observed in the OS in group 3 (log-rank, χ^2^ = 9.211, *P* = .002) and 4 (log-rank, χ^2^ = 8.732, *P* = .003, Fig. 3), respectively. Although no statistical differences were noticed in the OS between the other groups.

### 3.3 Subgroup analysis of recurrence and OS

For the subgroup analysis, several factors were entered into the Cox regression analysis to screen the factors that may affect the recurrence and survival, including times of adjuvant TACE, liver cirrhosis, AFP level (<400 or ≥400), tumor size (≤5 or >5), tumor number (1/2), Edmondson grade (I–-IV), and MiVI. Multivariate analysis showed 4 factors, including times of adjuvant TACE (HR = 0.797, 95%CI: 0.707–0.897, *P* < .001), tumor size (HR = 0.649, 95%, CI: 0.484–0.871, *P* = .004), Edmondson grade (Edmondson grade: HR = 0.563, 95%, CI: 0.423–0.750, *P* < .001), and MiVI (HR = 0.240, 95%, CI: 0.155–0.373, *P* < .001), were the risk factors for the recurrence. Meanwhile, these factors were also the risk factor of decreased survival duration (times of adjuvant TACE: HR = 0.523, 95%CI: 0.411–0.666, *P* < .001; tumor size: HR = 0.434, 95%, CI: 0.261–0.719, *P* = .001; Edmondson grade: HR = 0.317, 95%, CI: 0.193–0.521, *P* < .001; MiVI: HR = 0.137, 95%, CI: 0.072–0.259, *P* < .001). Although number of tumor lesions and cirrhosis maybe factors that potentially affect survival (tumor number: HR = 0.499, 95%, CI: 0.258–0.968, *P* = .04; cirrhosis: HR = 0.323, 95%, CI: 0.150–0.693, *P* = .004) rather than recurrence, these factors were not involved as stratum factors in our study due to the limitation of case number (Table 2). Then a stratified analysis was performed based on the tumor diameter to identify the effects on the recurrence or death. A low risk factor for recurrence or death was defined as Edmondson grade I/II without MiVI, while a high risk factor was defined as Edmondson grade III/IV or with MiVI. The number of patients with a tumor diameter of ≤5 cm in the low-risk subgroup was 66, 24, 24, and 29, respectively. Meanwhile, in patients with tumor diameter of ≤5 cm in the high-risk subgroup, the number was 32, 12, 11, and 10, respectively. In tumor diameter >5 cm low-risk subgroup, the number was 26, 14, 7, and 11, and in tumor diameter >5 cm high-risk subgroup, the number was 22, 10, 9, and 13, respectively.

**Table 2:**
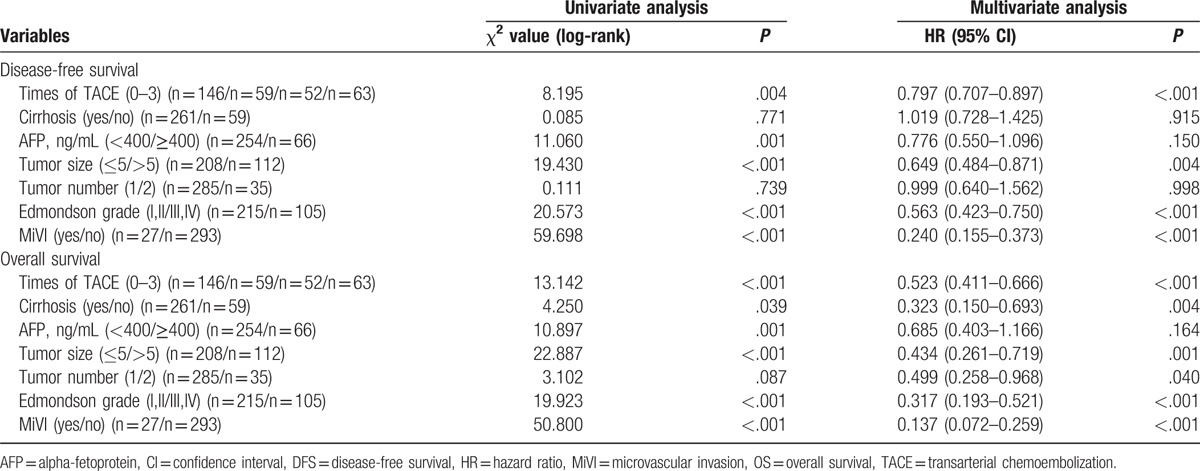
Uni- and multivariate analyses of DFS and OS.

**Figure 4:**
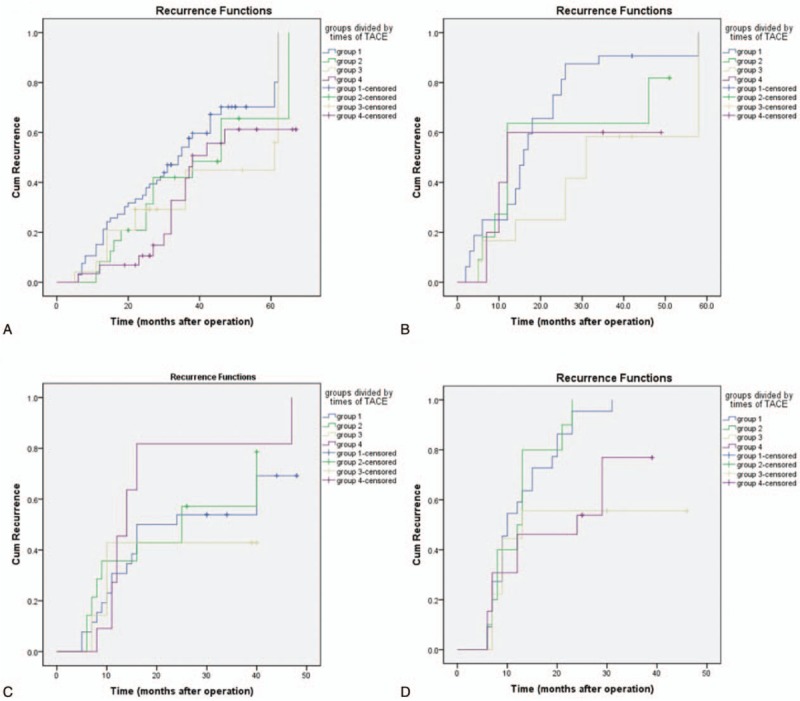
Recurrence curves for the subgroup analysis. (A) In the patients of low-risk subgroup with a tumor diameter of ≤5 cm, no statistical differences (log-rank, χ^2^ = 5.710, *P* = .127) were found between all the groups. (B) In patients of high risk group with a tumor diameter of ≤5 cm, statistical difference was found between group 3 and group 1 (log-rank, χ^2^ = 7.067, *P* = .008). (C) In the patients of low-risk subgroup with a tumor diameter of >5 cm, no statistical differences (log-rank, χ^2^ = 2.873, *P* = .412) were found between all the groups. (D) In the patients of high-risk subgroup with a tumor diameter of >5 cm, group 3 and 4 can delay recurrence (log-rank, χ^2^ = 4.212, *P* = .04; log-rank, χ^2^ = 5.642, *P* = .018).

**Figure 5:**
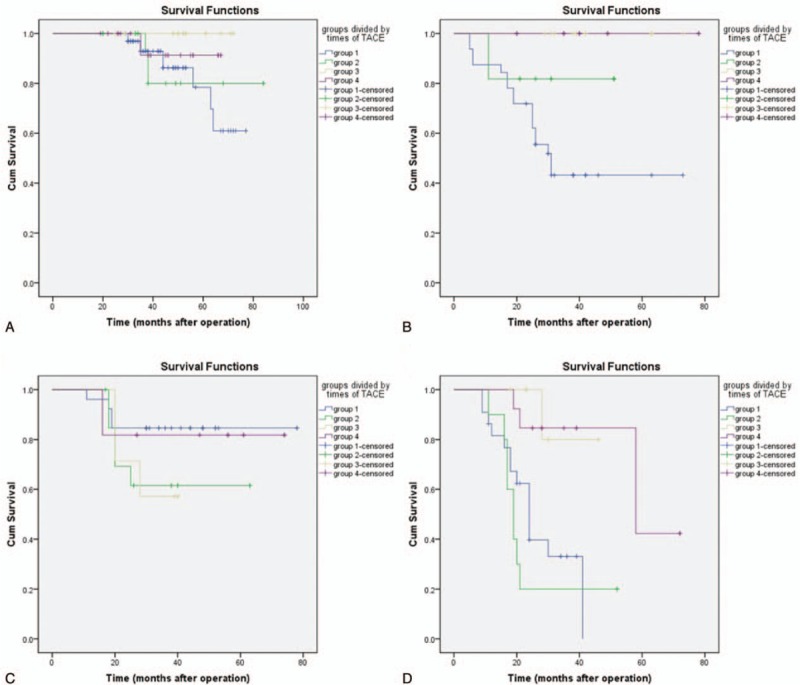
Survival curves of subgroups. (A) In the patients of low-risk subgroup with a tumor diameter of ≤5 cm, no statistical differences (log-rank, χ^2^ = 4.371, *P* = .224) were found between all the groups. (B) In the patients of high-risk subgroup with a tumor diameter of ≤5 cm, group 3 and 4 can improve overall survival (OS) compared to group 1 (group 3–1: log-rank, χ^2^ = 9.492, *P* = .002; group 4–1: log-rank, χ^2^ = 7.204, *P* = .007). (C) In the patients of low-risk subgroup with a tumor diameter of >5 cm, no significant statistical differences (log-rank, χ^2^ = 3.022, *P* = .388) were found between all the groups. (D) In the patients of high-risk subgroup with a tumor diameter of >5 cm, group 3 and 4 can improve OS compared to both group 1 and 2 (group 3–1: log-rank, χ^2^ = 6.186, *P* = .013; group 4–1: log-rank, χ^2^ = 8.748, *P* = .003; group 3–2: log-rank, χ^2^ = 8.370, *P* = .004; group 4–2: log-rank, χ^2^ = 11.526, *P* = .001).

Subgroup analysis showed that in low-risk subgroup regardless of the tumor diameter, no statistical differences were noticed in both DFS and OS (*P* > .05). In the patients with a tumor diameter of ≤5 in the high-risk subgroup, the recurrence was delayed in group 3 compared with group 1 (log-rank, χ^2^ = 7.067, *P* = .008). In addition, compared with group 1, the OS showed remarkable increase in group 3 (log-rank, χ^2^ = 9.492, *P* = .002) and 4 (log-rank, χ^2^ = 7.204, *P* = .007), respectively. In patients with a tumor diameter of >5 cm in the high-risk subgroup, the recurrence was delayed in group 3 (log-rank, χ^2^ = 4.212, *P* = .04) and 4 (log-rank, χ^2^ = 5.642, *P* = .018) compared with group 1. No statistical difference was noticed in the recurrence in the group 4 compared with group 2 (log-rank, χ^2^ = 3.946, *P* = .47). For the OS, compared with group 1, remarkable elevation was noticed in the OS in group 3 (log-rank, χ^2^ = 6.186, *P* = .013) and 4 (log-rank, χ^2^ = 8.748, *P* = .003), respectively. Meanwhile, compared with group 2, remarkable elevation was noticed in the OS in group 3 (log-rank, χ^2^ = 8.370, *P* = .004) and 4 (log-rank, χ^2^ = 11.526, *P* = .001), respectively (Figs. 4 and 5).

## 4 Discussion

Surgical resection is recommended for treating HCC patients with a single nodule,^[15]^ even resection in patients with intermediate-advanced HCC has been reported with satisfactory short- and long-term outcomes.^[16]^ However, the treatment outcome is highly affected by the HCC recurrence after resection. Increasing evidence reveals that TACE after radical resection of primary HCC is beneficial for the treatment outcome. For example, postoperative adjuvant TACE treatment can help to eliminate small residual cancer, reduce recurrence and prolong survival.^[17]^ On this basis, postoperative adjuvant TACE was reported to prolong the survival of high-risk patients with a tumor diameter of >5 cm, multiple nodules, or vascular invasion.^[18]^ In a prospective study involved HCC patients with vascular invasion or intrahepatic metastases, postoperative TACE was reported to improve the survival despite it showed no effects on the OS.^[19]^ Up to now, the potential benefits of adjuvant therapy and postoperative TACE in treating HCC are still not well defined due to presence of different entry criteria.

The development of HCC is a multistage process. A larger tumor size or poor differentiation was considered to affect the malignant biological characteristics of HCC.^[20,21]^ Some studies also demonstrated that MiVI was a major independent prognostic factor for HCC.^[22,23]^ Additionally, another study focused on recurrence of HCC after liver transplantation showed that poor tumor grading, MiVI, and tumor diameter were significant predictors of recurrence.^[24]^ The presence of MiVI was common in clinical practice, even for those with a tumor nodules of less than 5 cm.^[25]^ Nevertheless, it is still unknown whether these patients may benefit from the postoperative TACE after radical resection.

In the subgroup analysis including the potential risk factors such as tumor size, tumor differentiation, and MiVI, no statistical differences were found among all the low-risk subgroups, even for those with tumor size of more than 5 cm. According to the previous studies, postoperative TACE can cause progressive liver atrophy or hepatic insufficiency and liver function impairment.^[26,27]^ On this basis, we thought that postoperative TACE should not be abused in these patients. Although in the high-risk subgroups, patients received 2 or 3 postoperative TACE showed better DFS and OS compared to those received no or 1 TACE. This may be related to the fact that postoperative can detect and eliminate the residual cancer cells related to poor differentiation or MiVI that micrometastase via the bloodstream before or during liver resection. What is more, most of the blood supply to the HCC was derived from the hepatic artery. Recurrent tumors often occurred near the surgical resection margin.^[28,29]^ The postoperative TACE triggered the accumulation of chemotherapy agents to the local margins with increased blood supply to a healing wound, which contributed to the outcomes of the patients with these risk factors.

Although in our study, we showed that postoperative TACE as an adjuvant therapy could ameliorate the outcomes of patients with high risk factors such as poor histological differentiation and MiVI, there are limitations indeed. Take the therapy in the perspective of liver transplantation for an example, a former research showed that TACE as an pre-LT therapy failed to be predictive to recurrence of HCC.^[30]^ Therefore, further studies are needed to identify the value of TACE as an adjuvant or neoadjuvant way in radical procedures such as liver transplantation or radical resection of HCC. Meanwhile, the number of patients in each group and subgroup were not that sufficient, which may have a certain impact on the results. For example, the exact DFS and OS in different subgroups were not obtained due to lacking of outcomes (eg, recurrence or death) at a certain time point. What is more, our study is a unicenter nonrandomized study. Second, the results of this study may not be used for patients with hepatitis C- or alcohol-related HCC as most of patients were hepatitis B-related with cirrhosis.^[31]^ What is more, a study showed other factors such as serum AFP level could serve as a strong predictor of recurrence in HCC.^[30]^ However, these findings were not reported in our study, which could be partly because of different standard of including criteria. In future, further studies are needed to investigate factors such as serum AFP level and cirrhosis on the influences of recurrence and survival.

In conclusion, postoperative adjuvant TACE does not appear to improve OS or reduce recurrence in patients with low risk factors associated with poor prognosis. Nevertheless, it seems to improve OS or reduce recurrence in patients with high risk factors such as poor differentiation and MiVI. In future, RCTs with larger sample size should be performed to investigate the effects of postoperative TACE on HCC outcomes.
